# Protecting the most vulnerable: The urgent need to include HIV-exposed children in malaria chemoprevention strategies

**DOI:** 10.1371/journal.pmed.1004498

**Published:** 2024-12-10

**Authors:** Raquel González, Tacilta Nhampossa, Antía Figueroa-Romero, André-Marie Tchouatieu, Christine Manyando, Clara Menendez

**Affiliations:** 1 ISGlobal, Barcelona, Spain; 2 Centro de Investigação em Saúde de Manhiça (CISM), Maputo, Mozambique; 3 Consorcio de Investigación Biomédica en Red de Epidemiología y Salud Pública (CIBERESP), Madrid, Spain; 4 Facultat de Medicina i Ciències de la Salut, Universitat de Barcelona (UB), Barcelona, Spain; 5 Instituto Nacional de Saúde, Ministério de Saúde, Mozambique; 6 Medicines for Malaria Venture (MMV), Geneva, Switzerland; 7 Tropical Diseases Research Centre, Ndola, Zambia

## Abstract

Raquel González and colleagues discuss the drugs available to HIV-exposed children to prevent malaria infection and the urgent need to evaluate alternative agents.

**Summary points**

- Perennial malaria chemoprevention (PMC) is recommended for children living in high transmission malaria endemic countries.
- Sulfadoxine-pyrimethamine (SP), the first drug used for PMC, is contraindicated in individuals receiving a sulfa-based medication, including cotrimoxazole (CTX), due to possible sulfonamide-induced adverse drug reactions.
- Despite HIV-exposed children having a higher risk of severe malaria than HIV-unexposed children, they are not eligible to receive PMC with SP because they are usually on daily CTX prophylaxis.
- Thus, the most vulnerable children to malaria are currently the least protected by malaria chemoprevention strategies.
- Lessons learnt from malaria prevention in HIV–positive pregnant women show the need to evaluate alternative antimalarial drugs for PMC than can be safely administered to HIV-exposed children on CTX prophylaxis.

## Who are the children most vulnerable to malaria?

The burden of malaria and HIV infection overlap in sub-Saharan Africa, where it is estimated that 94% of malaria cases and 95% of malaria-related deaths occurred in 2022 [1]. The 2 diseases also share social and biological determinants of risk, being children one of the most susceptible populations to severe malaria [2].

In sub-Saharan Africa, HIV prevalence among pregnant women ranges from 2% to 30%, and more than 1 million women living with HIV become pregnant each year in the region [3,4]. Of note, the high prevalence of HIV infection and access to HIV mother-to-child transmission prevention programmes is leading to large numbers of HIV-uninfected children being born to women living with HIV in the region. It is estimated that annually at least 1 million babies are born HIV-exposed in malaria endemic areas. Of note, HIV-exposed children have increased morbidity and mortality in early childhood compared with children born to uninfected women [5]. Moreover, both HIV exposure and HIV infection have been associated with increased prevalence of severe malarial anemia during acute *Plasmodium falciparum* infection [6].

## Perennial malaria chemoprevention guidelines for children

In 2010, the World Health Organization (WHO) recommended intermittent preventive treatment in infants (IPTi) for malaria control in children living in areas with moderate-to-high malaria transmission [7]. This strategy consisted of the administration of a full therapeutic course of sulfadoxine-pyrimethamine (SP), whether or not parasites are present, through the Expanded Programme on Immunization at defined intervals corresponding to routine vaccination contacts at 10 weeks, 14 weeks, and 9 months of age [7]. In 2022, the WHO updated its chemoprevention recommendations and renamed IPTi as perennial malaria chemoprevention (PMC) [8]. The updated recommendation on PMC does not strictly specify the age groups targeted, given the evidence showing health benefits of malaria chemoprevention in children aged 12 to 24 months [8]. Sierra Leone was the first country to implement PMC as malaria control strategy nationwide in 2018. Since 2021, an increasing number of countries in sub-Saharan Africa have adopted the strategy and are starting its implementation [9,10].

SP, the first drug recommended for PMC, is contraindicated in individuals receiving a sulfa-based medication, including cotrimoxazole (CTX), due to possible sulfonamide-induced adverse drug reactions such as Stevens–Johnson syndrome, erythema multiforme, and leukopenia [11].

## Prevention of opportunistic infections in people living with HIV

CTX is a fixed-dose drug combination of trimethoprim and sulfamethoxazole which is recommended as daily prophylaxis in individuals with HIV to prevent opportunistic infections including malaria in settings with high prevalence of bacterial infections [12]. HIV-exposed children are also part of this recommendation and take daily CTX starting at 6 weeks of age until cessation of breastfeeding and exclusion of HIV infection [13]. This recommendation followed studies in adults living with HIV showing that CTX prophylaxis significantly reduced morbidity by preventing opportunistic infections [14]. Subsequent studies reported that CTX prophylaxis reduced morbidity in HIV–positive and in HIV-exposed uninfected children [15–20]. CTX has proven to also have antimalarial activity [21–23]. Based on this antimalarial effect of CTX, people living with HIV in malaria endemic countries are recommended the prophylaxis. However, coverage and adherence to the daily regimen constitute challenges for its effectiveness [24].

## A public health paradox and a “déjà vu”

Despite HIV-exposed children having a higher risk of severe malaria than HIV-unexposed children, they are not eligible to receive PMC with SP [5]. Thus, paradoxically, the most vulnerable children, those who are under 2 years of age and HIV-exposed, are currently the least protected against malaria by chemoprevention strategies. This constitutes a “déjà vu” regarding malaria preventive tools as it is the case of pregnant women living with HIV, since they also receive CTX prophylaxis for the same purpose [25]. Women with HIV are more susceptible to severe malaria and its related adverse pregnancy outcomes than HIV-uninfected women, but they cannot receive SP as intermittent preventive treatment. In spite of intermittent preventive treatment in pregnancy being recommended by the WHO since 1998, this strategy does not include HIV–positive women on CTX prophylaxis and consequently, they have been left out of this malaria prevention strategy for over 25 years [26]. In the last decades, it has been shown that adding an effective antimalarial drug as intermittent preventive treatment in pregnancy to CTX prophylaxis significantly improves malaria prevention [27]. Recently completed clinical trials evaluating dihydroartemisin-piperaquine for intermittent preventive treatment in pregnant women with HIV receiving CTX prophylaxis have showed a good antimalarial efficacy and safety of dihydroartemisin-piperaquine in this group [28,29]. This has led the WHO to finally consider dihydroartemisin-piperaquine as intermittent preventive treatment in pregnancy in women living with HIV.

## Way forward

The recently recommended malaria vaccines and other control strategies such as the insecticide-treated nets are targeted to all children living in stable malaria transmission areas regardless of their HIV status and exposure. In contrast, PMC with SP cannot be given to children receiving daily CTX. Lessons learnt from malaria prevention in HIV–positive pregnant women show the urgent need to evaluate alternative antimalarial drugs for PMC than can be safely administered to HIV-exposed children on CTX prophylaxis. Another recommended chemoprevention strategy, seasonal malaria chemoprevention is also based on a drug combination that includes SP, and therefore, HIV–positive children on CTX prophylaxis are being excluded to receive it. The currently renewed implementation efforts of malaria chemoprevention tools should address all population groups and focus on those groups that are most vulnerable to the infection such as HIV–positive children and pregnant women.

When addressing the potential antimalarial candidate to add to CTX for PMC the following characteristics should be considered: (i) having a long half-life; (ii) availability of a pediatric formulation (ideally dispersible) to optimize infants drug intake; (iii) being safe and well tolerated; (iv) retaining a good antimalarial efficacy; and (v) low risk of *Plasmodium* drug resistance development. Other important considerations are costs and availability of the manufactured drug. Table 1 summarizes the properties of potential antimalarial drugs that could be used for PMC in children on CTX prophylaxis. Amodiaquine and dihydroartemisin-piperaquine are the drugs that offer the best advantages for this purpose since they have a long have life and a pediatric formulation is available. Of note, there are no reported drug–drug interactions between dihydroartemisin-piperaquine and dolutegravir-based regimens, which is the first line antiretroviral therapy for children living with HIV [2]. Regarding amodiaquine, a study evaluating drug–drug interactions between dolutegravir and artesunate-amodiaquine, reported a decrease in dolutegravir concentrations but of unlikely clinical significance [30]. Amodiaquine concentrations could also be reduced when administered concomitantly with nevirapine, but this reduction is unlikely to be of clinical relevance [2]. On the other hand, in some malaria endemic countries dihydroartemisin-piperaquine is one of the first line treatment of uncomplicated malaria episodes, and there is concern regarding the use of the same drug for treatment and prevention because of the potential increased risk of development of drug resistance by the parasite.

**Table 1:** Characteristics of antimalarial drugs candidates for PMC.

	Atovaquone-Proguanil	Mefloquine	Dihydroartemisin-piperaquine	Artesunate-Amodiaquine	Amodiaquine
Pediatric Formulation	Pediatric ND^1^	No	Dispersible	Pediatric ND	Dispersible
Price (tablet)	>2 USD	>2 USD	<1 USD	<0.5 USD	<0.5 USD
Half-life^2^	Short	Long	Long	Long	Long
Posology/Regimen	Daily	Single day	3-day	3-day	3-day
Tolerability	Poor^3^	Poor^6^	Good^8^	Good^10^	Good^12^
Efficacy	Good^4^	Good^6^	Good^8^	Good^10^	Good^12^
Risk of Resistance	Low^5^	Low^7^	Moderate^9^	Moderate^11^	Low^13^

^1^Pediatric ND: pediatric presentation available but not dispersible.

^2^Long: over 10 days.

^3^Lell and colleagues, Lancet 1998.

^4^Blanshard and colleagues, The Cochrane database of systematic reviews, 2021.

^5^David and colleagues, J Infect Dis, 2003.

^6^Faye and colleagues, Am J Trop Med Hyg, 2010.

^7^Aubouy and colleagues, Trop Med Int Health, 2007.

^8^Kamya and colleagues, AIDS, 2014.

^9^Gupta and colleagues, PLoS One, 2020.

^10^Odhiambo and colleagues, PLoS One, 2010.

^11^Tumwebaze and colleagues, Lancet Microbe, 2021.

^12^ Massaga and colleagues, Lancet, 2003.

^13^Grais and colleagues, Malaria J, 2018.

Research on the efficacy and safety of alternative drugs that could be administered concomitantly with CTX for PMC and seasonal malaria chemoprevention in HIV-exposed and HIV–positive children is needed. This should ideally include randomized placebo-controlled trials to evaluate the candidate antimalarial drug efficacy in settings with different malaria transmission intensities. The acceptability and cost-effectiveness of the PMC drug candidate should also be assessed in order to inform its potential implementation.

In conclusion, decades of experience in the evaluation of malaria prevention with intermittent preventive treatment in pregnancy for women living with HIV on CTX prophylaxis should be a lesson to not leave their infants unprotected against malaria. Currently, these HIV-exposed infants remain deprived from recommended effective malaria chemoprevention strategies, which is an unacceptable inequity in global health that should be urgently addressed. Clinical and epidemiological research of alternative antimalarial drugs for PMC should guide the development of malaria control tools that include the most vulnerable children to the infection.

## Abbreviations

CTX: cotrimoxazole
IPTi: intermittent preventive treatment in infants
PMC: perennial malaria chemoprevention
SP: sulfadoxine-pyrimethamine
WHO: World Health Organization
